# Gouty tophi in the thoracic spinal canal with incomplete paraplegia: a case report

**DOI:** 10.3389/fsurg.2025.1708430

**Published:** 2025-12-15

**Authors:** Chunyu Zhao, Juncai Deng, Jingzhe Ding, Kunpeng Zhang, Jian Zhang

**Affiliations:** 1 School of Clinical Medicine of Chengdu University of TCM, Chengdu University of Traditional Chinese Medicine, Chengdu, China; 2 Department of Spinal Surgery, Chengdu Pidu District Hospital of Traditional Chinese Medicine, Chengdu, China

**Keywords:** paraplegia, spinal canal, gouty tophi, case report, orthopedic surgery

## Abstract

Gout manifesting in the spine is a relatively rare occurrence. Involvement of the lumbar spine is more common in cases of spinal gout, while reports of gouty tophi forming within the thoracic spinal canal are exceedingly rare. This article reports a case of incomplete paraplegia caused by gouty tophi in the thoracic spinal canal. The patient, a 56-year-old man, returned to normal after undergoing surgical treatment and was monitored for ten months postoperatively. This case highlights the importance of early diagnosis of spinal gout to prevent the progression of neurological symptoms. Surgical treatment for paraplegia caused by intraspinal tophi can yield favorable clinical outcomes. Increasing awareness of spinal gout and implementing effective uric acid management strategies are essential for preventing the severe consequences associated with this condition.

## 1 Introduction

Gout, a common disease, typically affects both articular and nonarticular structures and is caused by the deposition of monosodium urate (MSU) crystals (1). Tophi develop in cases of untreated or uncontrolled gout (2). The initial attack of gout is usually characterized by inflammatory arthritis in the lower extremities, whereas spinal involvement is relatively uncommon (3). In patients with spinal gout, the lumbar spine is the most frequently affected area (4). However, cases involving the thoracic spine that leads to the formation of intraspinal tophi and results in paralysis are extremely rare. We admitted and treated a patient with tophi located in the thoracic spinal canal, which resulted in incomplete paraplegia. Our intervention yielded favorable outcomes.

## 2 Case presentation

**Figure 1:**
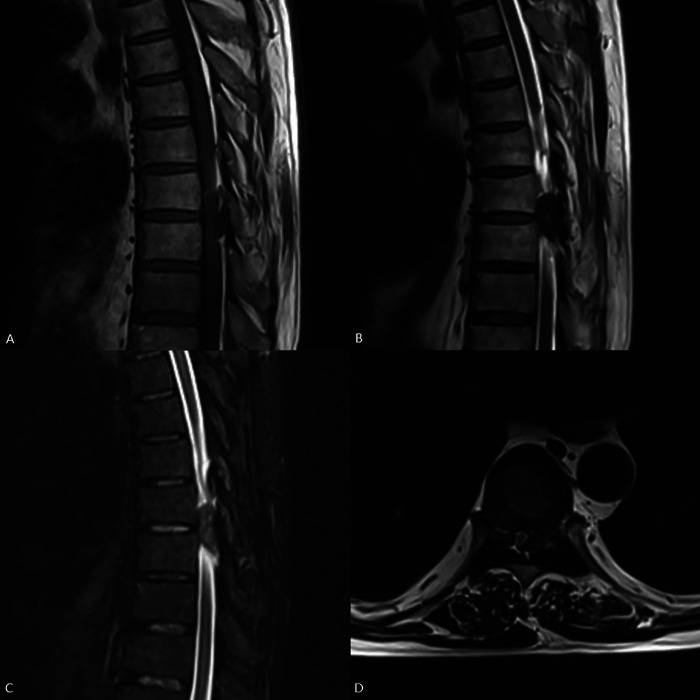
Thoracic spinal epidural lesion on MRI: **(A)** T1-weighted imaging (T1WI), **(B)** T2-weighted imaging (T2WI), **(C)** T2-weighted fat-suppressed imaging (T2W-FS), **(D)** T2WI transverse axis view. An irregular lesion exhibiting long T1 and short T2 signal intensity is observed in the epidural space of the thoracic spinal canal at the T8-T9 levels. The lesion demonstrated isointensity on T2W-FS. Spinal cord compression is evident.

**Figure 2:**
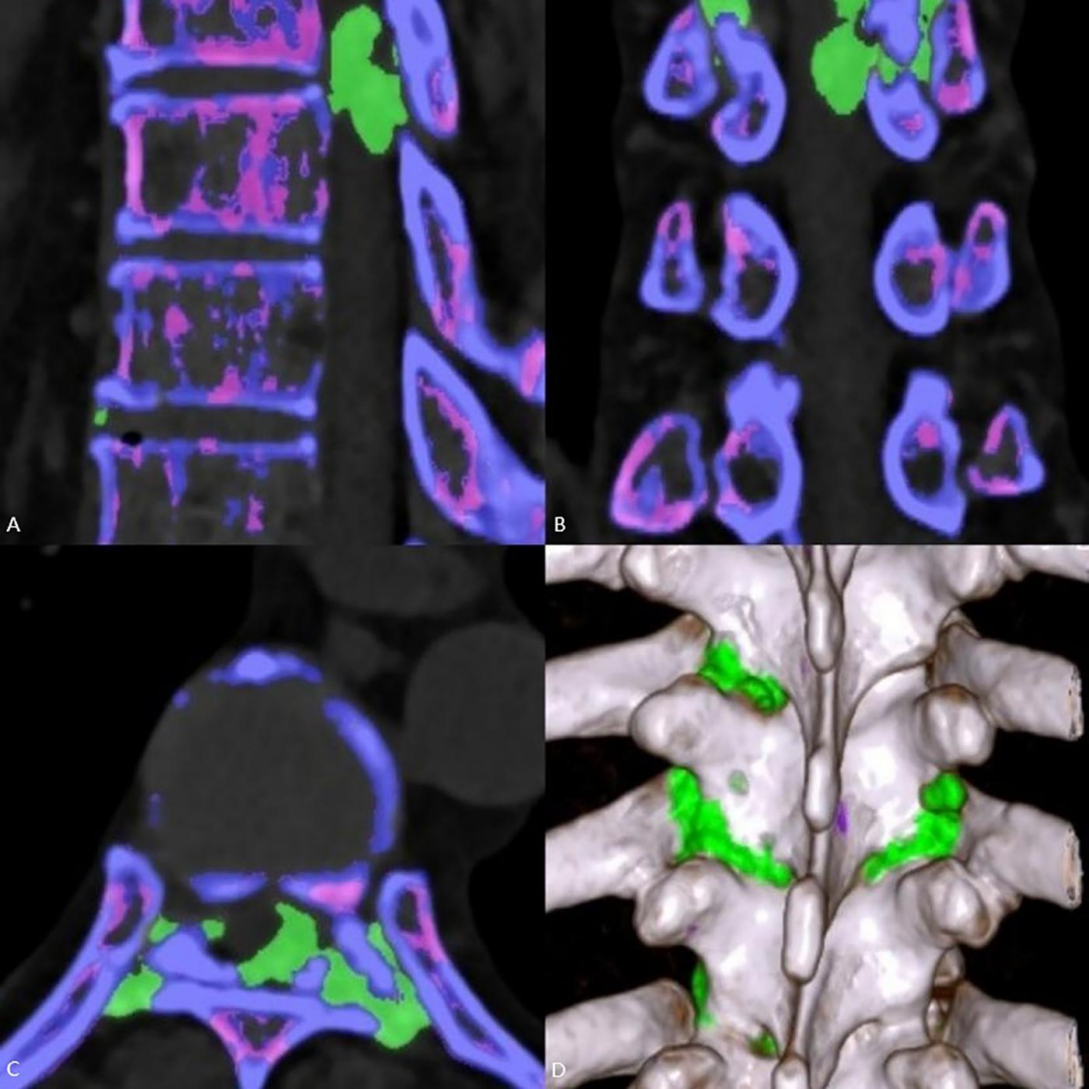
DECT imaging: **(A)** sagittal view, **(B)** coronal view, **(C)** transverse view, **(D)** 3D reconstruction. Mottled and nodular green pseudocolor coding, which indicates urate deposition, is observed in the following locations: the margins of the thoracic vertebral bodies and articular processes; the left interlaminar spaces of T7–T10; the right interlaminar spaces of T8–T9; and nodular deposits within the spinal canal.

**Figure 3:**
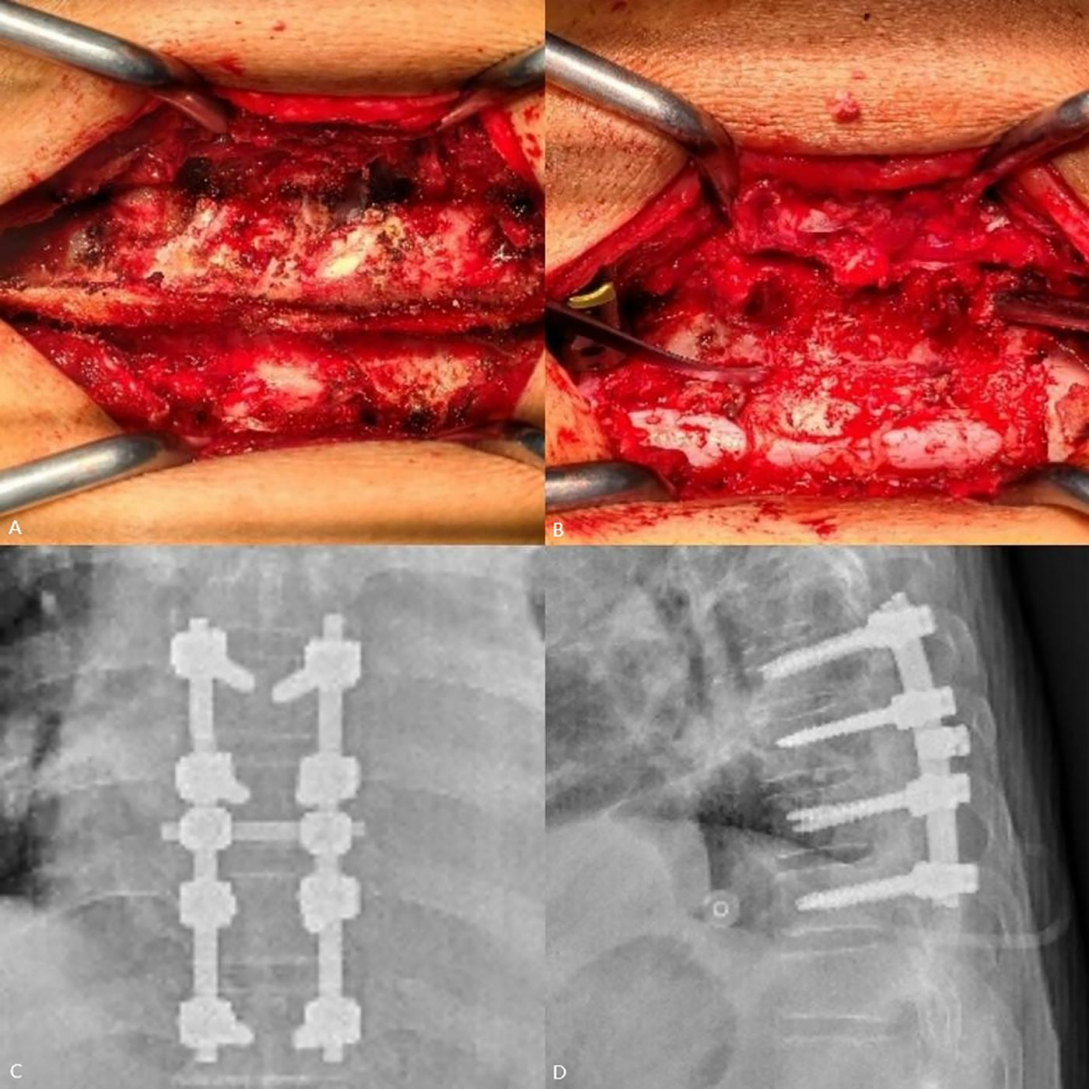
Intraoperative photos and postoperative imaging **(A)** intraoperative view: gypsum-like white particles (indicative of calcific or urate deposits) are present within the interlaminar space (indicated by the arrow). **(B)** Postdecompression: Gypsum-like white particle deposits in the left spinal canal cause spinal cord compression and deformation (indicated by the arrow). **(C,D)** Postoperative anteroposterior and lateral x-rays: demonstrating adequate decompression and restoration of spinal canal integrity.

**Figure 4:**
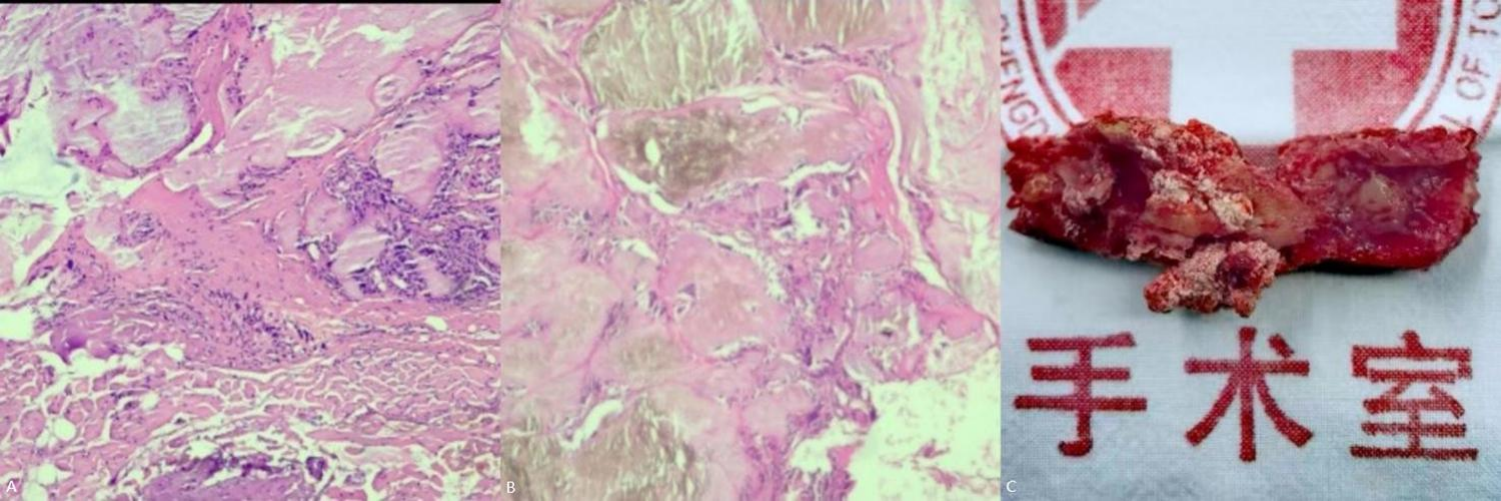
Pathological and surgical specimens: **(A)** photomicrograph outside the spinal canal; **(B)** photomicrograph inside the spinal canal; **(C)** appearance of the vertebral lamina following *en bloc* resection. Gypsum-like white particle is MSU.

A 56-year-old man presented with weakness in both lower extremities that had begun two weeks prior. One week prior to his hospital visit, he fell while walking and subsequently developed numbness in the soles of both feet, as well as urinary and fecal incontinence. This prompted him to seek medical attention. He has a 10-year history of hypertension and hyperuricemia. Two years ago, he developed tophi in several areas of his body and began experiencing chronic back pain. One year later, the patient underwent staged surgery to remove the tophi from both hands. Examination following admission revealed numbness below the groin level, muscle strength in both lower extremities at approximately Grade 4, positive ankle clonus, weakened anal sphincter contraction, and an absent cremasteric reflex, ASIA Impairment Scale (AIS) grade was D. The serum uric acid (UA) level was measured at 519.70 µmol/L. MRI revealed epidural lesions at the T8–T9 thoracic spinal levels, accompanied by spinal cord compression (Figure 1). Given the patient's long-standing history of gout, dual-energy CT (DECT) was performed, which confirmed the deposition of MSU within the lesions (Figure 2). One week later, the serum UA level was 382.8 µmol/L. The patient subsequently underwent surgical intervention, which included spinal canal decompression, lesion removal, pedicle screw fixation, and posterolateral bone grafting using artificial bone (Figure 3). After the operation, the patient's numbness below the groin level was alleviated, and the strength of the key muscles in the lower extremities was assessed at Grade 4. Additionally, the patient participated in rehabilitation training. Two weeks after surgery, the surgical incision had healed, and the stitches were removed. Bowel and bladder function, perineal sensation, and muscle strength in both lower extremities returned to normal, AIS grade was E. Febuxostat was subsequently administered orally. Postoperative pathological examination confirmed that the lesions both inside and outside the spinal canal were tophi (Figure 4). After discharge, the patient continued to take febuxostat orally and was monitored for ten months. The results of the neurological examination were normal, and the serum UA levels remained within the normal range.

## 3 Discussion

Gout is an inflammatory arthritis caused by the deposition of MSU crystals in the synovial fluid and cartilage. These deposits can be widely distributed across various tissues throughout the body, including the facet joints of the axial skeleton and the intervertebral discs (5). Hyperuricemia is recognised as a critical factor in the formation of gouty tophi; however, studies have shown that approximately 16.8% of spinal gout cases occur in the absence of hyperuricemia or any prior history of gout (6). Furthermore, the formation of MSU crystals is influenced by factors such as low temperature, pH (within the range of 7–9), and elevated sodium ion concentrations (7–9). Additionally, joints that are damaged or affected by osteoarthritis are more susceptible to crystal deposition (9–11). Kersley et al. (12) first reported on spinal gout in 1950, and since then, additional case reports have been published by other researchers. A systematic review of Medline and EMBASE, conducted from inception to April 15, 2023, identified a total of 315 patients with spinal gout. The lumbar spine was the most commonly affected spinal segment. The most common symptom is back pain, whereas paraplegia and quadriplegia are observed in only 14 cases (4). It has been suggested that, compared with patients without axial gout, factors such as disease duration, age, serum UA levels, and hypertension do not correlate with the presence of axial gout. In contrast, the presence of diabetes has been associated with spinal gout (4, 5). However, our patient, who had a 10-year history of hypertension and hyperuricemia, developed multiple tophi two years prior and experienced chronic back pain. A review of the patient's medical history, revealed that intraspinal tophi development was due to an inadequate understanding of gout and poor management of serum UA levels.

Although early diagnosis of spinal gout is crucial for preventing the progression of neurological symptoms, achieving this diagnosis can be challenging. Conventional imaging techniques, such as x-ray and MRI, often fail to provide definitive evidence. However, DECT can be advantageous in differentiating spinal gout from other conditions (13). The diagnosis of gout typically relies on polarized light microscopy to identify MSU crystals in synovial fluid. However, this method has several limitations, including potential complications, a high rate of false negatives, and the inability to aspirate MSU crystals that may be deposited in soft tissues. DECT has emerged as a highly sensitive and minimally invasive alternative, offering several advantages. It can visualize both intra-articular and extra-articular MSU deposits and monitor changes in crystal burden over time (14). The American College of Rheumatology and the European League Against Rheumatism formally incorporated the presence of urate deposition, as demonstrated by DECT, into the 2015 gout classification criteria (15). The patient had a history of long-standing gouty arthritis and multiple tophi distributed throughout the body, which raised our suspicion of tophi in the spinal canal lesions. A subsequent DECT examination revealed that the lesions were consistent with tophi. Postoperative histopathological examination further confirmed the presence of MSU crystals in the spinal canal.

The first-line treatment for patients with spinal gout typically involves urate-lowering therapy and conservative management with anti-inflammatory agents (16). If progressive neurological deficits or new neurological symptoms develop, surgical resection and decompression are generally indicated; these procedures have a very low incidence of postoperative complications (4). In this case, a preoperative diagnosis of intraspinal tophi was established, and symptoms of paraplegia had already manifested. Given the clear indications for surgery, an intervention was performed. Importantly, owing to the large size of the lesion within the spinal canal and the severe compression of the spinal cord, a long-segment, *en bloc* resection of the tissue behind the spinal canal was necessary to prevent further damage to the spinal cord.

However, paraplegia or quadriplegia resulting from spinal gout, although rare, is a serious condition that presents ongoing challenges in diagnosis and management, including high rates of preoperative misdiagnosis and a lack of standardised treatment strategies. To thoroughly elucidate the clinical features and evaluate the effectiveness of diagnostic and therapeutic approaches in this case, we retrieved, reviewed, and analysed 31 cases of spinal gout-induced paraplegia or tetraplegia from the PubMed database, followed by a comparative analysis. The reports comprised 24 male (17–40) and 7 female patients (41–47), a gender distribution consistent with that of systemic gout (48). Preoperative diagnosis primarily relied on MRI and/or conventional CT. A definitive diagnosis was achieved preoperatively in only one case via CT-guided percutaneous biopsy (45), and DECT was employed in another case (37). The preoperative misdiagnosis rate was high [14 out of 31 cases were misdiagnosed as tumor, infection, or other conditions (17, 21, 25, 28, 29, 31, 32, 36, 38, 40, 42, 45–47)], with postoperative pathological examination serving as the mainstay for definitive diagnosis. A definitive preoperative diagnosis is essential for formulating an appropriate surgical plan. As a non-invasive technique to identify urate crystals, DECT plays a crucial role in the preoperative differential diagnosis of such conditions. Furthermore, monitoring UA levels is crucial for preventing acute gout flares during the perioperative period and recurrence within the 1-year follow-up (49). However, in the majority of these 31 case reports, the specific UA management strategies were not described in detail (18/31) (18, 20, 22–26, 31, 34–39, 41, 42, 46, 47), and follow-up information was missing in 13 cases (17, 20, 24, 26, 28, 29, 33, 35, 38, 39, 41, 43, 47). Patients with tophi are at an elevated risk of gout recurrence. It is advisable to maintain the preoperative serum UA level below 300 μmol/L (49). In this case, the patient had already developed paralysis, and surgical intervention was deemed necessary to address the paralytic symptoms promptly. After thorough consideration, uric acid-lowering therapy was initiated postoperatively. Current follow-up data indicate that the overall therapeutic effect is satisfactory; however, long-term follow-up is still needed.

## 4 Conclusion

The clinical manifestations of early-stage spinal gout are often atypical. When the disease is suspected, DECT can aid in differential diagnosis. Paraplegia caused by intraspinal tophi can be effectively managed through surgical treatment, resulting in favorable clinical outcomes. Enhancing awareness of spinal gout and optimizing the management of UA levels are crucial for preventing serious consequences.

## Data Availability

The original contributions presented in the study are included in the article/Supplementary Material, further inquiries can be directed to the corresponding author.
